# The Role of Indoleamine 2,3-Dioxygenase in Renal Tubular Epithelial Cells Senescence under Anoxia or Reoxygenation

**DOI:** 10.3390/biom11101522

**Published:** 2021-10-15

**Authors:** Theodoros Eleftheriadis, Georgios Pissas, Georgios Filippidis, Vassilios Liakopoulos, Ioannis Stefanidis

**Affiliations:** Department of Nephrology, Faculty of Medicine, University of Thessaly, 41110 Larissa, Greece; gpissas@msn.com (G.P.); gfilippid@yahoo.gr (G.F.); liakopul@otenet.gr (V.L.); stefanid@med.uth.rg (I.S.)

**Keywords:** senescence, indoleamine 2,3-dioxygenase, DNA damage response, p21, p16, ischemia-reperfusion, acute kidney injury

## Abstract

Ischemia-reperfusion injury is the commonest form of acute kidney injury (AKI). Tubular epithelial cell senescence contributes to incomplete recovery from AKI and predisposes to subsequent chronic kidney disease. In cultures of primary proximal renal tubular epithelial cells (RPTECs) subjected to anoxia or reoxygenation, we evaluated the role of indoleamine 2,3-dioxygenase 1 (IDO) in cellular senescence. Proteins of interest were assessed with Western blotting or enzyme-linked immunosorbent assay or histochemically. Under anoxia or reoxygenation, IDO expression and activity were increased. Moreover, the two IDO-derived pathways, the general control nonderepressible 2 kinase (GCN2K) pathway and the aryl-hydrocarbon receptor (AhR) pathway, were also activated. A DNA damage response (DDR) took place and led to increased levels of the cell-cycle inhibitors p21 and p16, and senescence-associated β-galactosidase (SA-β-Gal) activity. Cell proliferation was inhibited, and more IL-6 was produced. The IDO inhibitor 1-DL-methyl-tryptophan ameliorated the DDR; decreased p21, p16, and SA-β-Gal activity; restored cell proliferation; and decreased IL-6 production. The AhR inhibitor CH223191 did not affect the above parameters. In conclusion, anoxia and the subsequent reoxygenation upregulate IDO. IDO depletes tryptophan and activates GCN2K. The latter enhances the anoxia- or reoxygenation-induced DDR, resulting in increased p21 and p16 expression and eventually leading to RPTEC senescence. Since cellular senescence affects AKI outcome, the role of IDO in cellular senescence and the possible therapeutic role of IDO inhibitors deserve further investigation.

## 1. Introduction

Acute kidney injury (AKI) is becoming increasingly prevalent, especially among hospitalized patients [1]. Due to their high metabolic demands, renal tubular epithelial cells are extremely vulnerable to ischemia-reperfusion (I-R) injury, with the latter being the commonest cause of AKI [2]. I-R injury consists of two consecutive, yet distinct pathophysiological phases. During ischemia, energy deprivation induces cell injury. However, during reperfusion, reactive oxygen species (ROS) overproduction harms the cells [3]. The pathophysiological difference between the two phases of I-R injury becomes evident by the different cell-death types that prevail in each of them. Under ischemia, renal proximal tubular epithelial cell (RPTEC) death ensues through apoptosis [4], whereas under reperfusion, ferroptosis prevails [4,5,6].

Besides its direct effect on patient survival [1], AKI increases the possibility of progression to chronic kidney disease (CKD) [7]. Renal tubular epithelial cell senescence has been incriminated in the latter [8,9]. Senescent cells enter a state of permanent cell cycle arrest, and concurrently secrete a variety of proinflammatory and profibrotic cytokines [8,9]. I-R injury induces renal tubular epithelial cell senescence, likely through the DNA damage response (DDR) that occurs during such an insult [8,9,10]. Senescence contributes to the long-term consequences of AKI. Cellular senescence prevents renal tubular epithelial cell dedifferentiation and proliferation necessary for tubular regeneration and complete kidney recovery. In parallel, cytokines produced by the senescent cells promote inflammation and fibrosis [8,9,11]. Fibrosis is the hallmark of CKD, and administration of senolytics by inducing apoptosis of senescent tubular epithelial cells ameliorates renal fibrosis in a murine model of unilateral renal I-R injury [12].

Indoleamine 2,3-dioxygenase 1 (IDO) degrades tryptophan through the kynurenine pathway. By depleting tryptophan, IDO activates the general control nonderepressible-2 kinase (GCN2K), which alters the translational program of the cell [13,14]. Remarkably, except IDO-induced tryptophan depletion, GCN2K is also activated by various factors capable of inducing cellular senescence stress conditions, such as oxidative stress and ultraviolet irradiation [13,15]. Elegant studies showed that upon activation, GCN2K facilitates the DDR [16,17], which may lead to cell senescence or apoptosis [8,9,10]. In parallel, kynurenine, produced by tryptophan degradation, activates the transcription factor aryl-hydrocarbon receptor (AhR), altering the expression of many genes [18,19]. Interestingly, IDO is increased during I-R, whereas IDO inhibition decreases renal tubular cell death and facilitates kidney recovery [20]. In addition, in RPTECs, IDO is upregulated by anoxia and reoxygenation and promotes cell apoptosis under anoxic conditions and ferroptosis during reoxygenation [21]. However, the role of IDO in I-R-induced renal tubular epithelial cells senescence has not yet been assessed.

In the current study, we evaluated whether IDO contributes to renal tubular epithelial cell senescence. We developed an appropriate cell culture system to assess the effect of the two distinct phases of I-R injury on IDO kinetics and whether the latter affects RPTEC senescence. To imitate ischemia, RPTECs were cultured under anoxic conditions. To simulate reperfusion, RPTECs were initially cultured under anoxia, then washed and supplied with fresh culture medium, and normoxic conditions were applied. To evaluate the possible role of the two aforementioned IDO activity-induced molecular pathways on cellular senescence, the IDO inhibitor 1-DL-methyl-tryptophane (1-MT) or the AhR inhibitor CH223191 was used [22,23].

## 2. Materials and Methods

### 2.1. Cell Culture Conditions

Primary mouse RPTECs (cat. no. C57-6015, Cell Biologics, Chicago, IL, USA) were cultured in a Complete Epithelial Cell Medium/w kit, and supplemented with epithelial cell growth supplement (epithelial growth factor, insulin, transferrin, L-glutamine, selenium, fetal bovine serum, and antibiotics) (cat. no. M6621, Cell Biologics). Cells were initially cultured in 75 cm^2^ flasks, and passage three cells were used for the experiments.

For the experiments, a density of 300,000 cells per well in 6-well plates was used. Cells were incubated for 16 h before the onset of anoxic conditions. To reduce oxygen levels to less than 1%, the GasPak EZ Anaerobe Container System with Indicator (cat. no. 26001, BD Biosciences, S. Plainfield, NJ, USA) was used. Cells within the anaerobe container were cultured at 37 °C for 24 h. This time point was selected according to previous studies using the same type of cells and conditions [4,21]. These anoxic conditions imitate ischemia.

In reoxygenation experiments, cells previously cultured under anoxia were washed with Dulbecco’s phosphate buffer saline (PBS) (Sigma-Aldrich; Merck Millipore, Darmstadt, Germany), supplemented with fresh culture medium, and placed at 37 °C in a humidified atmosphere containing 5% CO_2_ for 2 h. This time point was selected according to previous studies using the same type of cells and conditions [4,21]. These conditions simulate reperfusion.

In the case of experiments with human cells, primary human RPTECs (cat. no. 4100, ScienCell, Carlsbad, CA, USA) were cultured under the same conditions as the mouse RPTECs. However, human RPTECs are more sensitive than mouse RPTECs in anoxia but less vulnerable to cell damage under reoxygenation [4]. Thus, these cells were subjected to anoxia for 2 h and to reoxygenation for 24 h.

Whenever needed, cells were treated with 100 μM of the IDO inhibitor 1-MT (Sigma-Aldrich; Merck Millipore) or 3 μM of the AhR inhibitor CH223191 (Sigma-Aldrich; Merck Millipore). The above concentrations were selected according to previous studies using the same type of cells and conditions [21,24]. In the anoxia experiments, the inhibitors were added at the onset of the anoxic conditions. In the reoxygenation experiments, the inhibitors were administered at the beginning of the reoxygenation in previously untreated cells that were subjected to anoxia.

### 2.2. Assessment of Proteins of Interest

Cultured RPTECs were lysed with the T-PER tissue protein extraction reagent (Thermo Fisher Scientific Inc., Waltham, MA, USA) supplemented with protease and phosphatase inhibitors (Sigma-Aldrich; Merck Millipore, and Roche Diagnostics, Indianapolis, IN, USA, respectively). After quantification with Bradford assay (Sigma-Aldrich; Merck Millipore), 10 μg from each protein extract were electrophoresed in sodium dodecyl sulfate (SDS) polyacrylamide gel (4–12% Bis-Tris gels, Thermo Fisher Scientific Inc.) and transferred onto a polyvinylidene fluoride (PVDF) membrane (Thermo Fisher Scientific Inc.). Western blot bands were detected with the LumiSensor Plus Chemiluminescent HRP Substrate kit (GenScript Corporation, Piscataway, NJ, USA). For reprobing the PVDF membranes, the Restore Western Blot Stripping Buffer (Thermo Fisher Scientific Inc.) was used. Densitometric analysis of the bands was performed with the Image J software version 1.53f (National Institute of Health, Bethesda, MD, USA). These experiments were repeated four times.

Blots were incubated at 4 °C for 16 h with each primary antibody and for 30 min at room temperature with the appropriate secondary antibody. Primary antibodies were specific for the following proteins: IDO (1:200, sc-25809, Santa Cruz Biotechnology, Dallas, TX, USA); GCN2K (1:100, cat. no sc-374609, Santa Cruz Biotechnology), phosphorylated at Thr899 GCN2K (p-GCN2K, 1:1000, cat. no ab75836; Abcam, Cambridge, UK); cytochrome P450, family 1, subfamily A, polypeptide 1 (CYP1A1, 1:500, cat. no. sc-25304, Santa Cruz Biotechnology); ataxia telangiectasia mutated kinase (ATM, 1:1000, cat. no 2873, Cell Signaling Technology, Danvers, MA, USA), phosphorylated at Ser1981 ATM (p-ATM, 1:1000, cat. no 5883, Cell Signaling Technology); p53 (1:1000, cat. no 2524, Cell Signaling Technology), phosphorylated at Ser15 p53 (p-p53, 1:1000, cat. no9284, Cell Signaling Technology); p21 Waf1/Cip1 (p21, 1:1000, cat. no 37543, Cell Signaling Technology); p16 INK4A (p16, 1:1000, cat. no 80772, Cell Signaling Technology), marker of proliferation Ki-67 (Ki-67, 1:1000, cat no. NBP2-22112, Novus Biologicals, Abingdon, Oxon, UK); and β-actin (1:2500, cat. no. 4967, Cell Signaling Technology). As secondary antibodies, the anti-mouse IgG, HRP-linked antibody (1:1000, cat. no 7076, Cell Signaling Technology), or the anti-rabbit IgG, HRP-linked antibody (1:1000, cat. no 7074, Cell Signaling Technology) were used. (Original Western blots are available as a supplementary file.)

### 2.3. Assessment of Tryptophan Catabolism through the Kynurenine Pathway and IL-6 Production

Tryptophan catabolism through the kynurenine pathway was assessed via the concentration in the cell culture supernatant. The ratio of kynurenine to tryptophan concentration corresponds to IDO activity. Tryptophan and kynurenine concentrations were measured with competitive enzyme-linked immunosorbent assay using the kynurenine/tryptophan ratio ELISA pack (cat. no ISE-2227, ImmuSmol, Bordeaux, France) on an EnSpire Multimode Plate Reader (Perkin Elmer, Waltham, MA, USA). The detection limits of the kit are 47.5 ng/mL for kynurenine and 1.2 μg/mL for tryptophan, respectively. These experiments were repeated six times.

Interleukin-6 (IL-6) production was assessed by its concentration in the cell culture supernatant. IL-6 was measured with the Mouse IL-6 ELISA kit (cat. no E-EL-M0044, Elabscience Biotechnology Inc., Houston, TX, USA) on an EnSpire Multimode Plate Reader. These experiments were repeated six times.

### 2.4. Assessment of Senescence-Associated β-Galactosidase Activity

Senescence-associated β-galactosidase (SA-β-Gal) activity was assessed histochemically using a commercially available kit (cat. no. 9860, Cell Signaling Technology). Mouse and human RPTECs were immediately fixed after the end of the anoxia or reoxygenation period and stained according to the manufacturer’s protocol except for the use of the double X-Gal reagent concentration. An inverted microscope (Axiovert 40C; Carl Zeiss AG, Göttingen, Germany) and a digital camera (3MP USB2.0 Microscope Digital Camera; AmScope, Los Angeles, CA, USA) with the related software (AmScope v. x64, 3.7.3036; AmScope) were used for cell imaging. The percentage of staining for SA-β-Gal activity to the total number of cells x100 was calculated. These experiments were repeated three times.

### 2.5. Statistical Analysis

The IBM SPSS Statistics for Windows, version 26 (IBM Corp., Armonk, NY, USA), was used for the statistical analysis. A one-sample Kolmogorov–Smirnov test verified the normal distribution of almost all the variables, and for comparison of means, the one-way analysis of variance with Bonferroni’s post hoc test was used. The Kruskal–Wallis H-test and Mann-Whitney U-test were used to analyze the histochemistry results since they were not normally distributed. The results are expressed as mean ± SEM, and statistical significance was set at a *p* < 0.05. Western blotting results were normalized for β-actin, and then for readers’ convenience, they are depicted after normalization for the control group.

## 3. Results

### 3.1. Anoxia and Reoxygenation Upregulate IDO Expression and Activity

**Figure 1:**
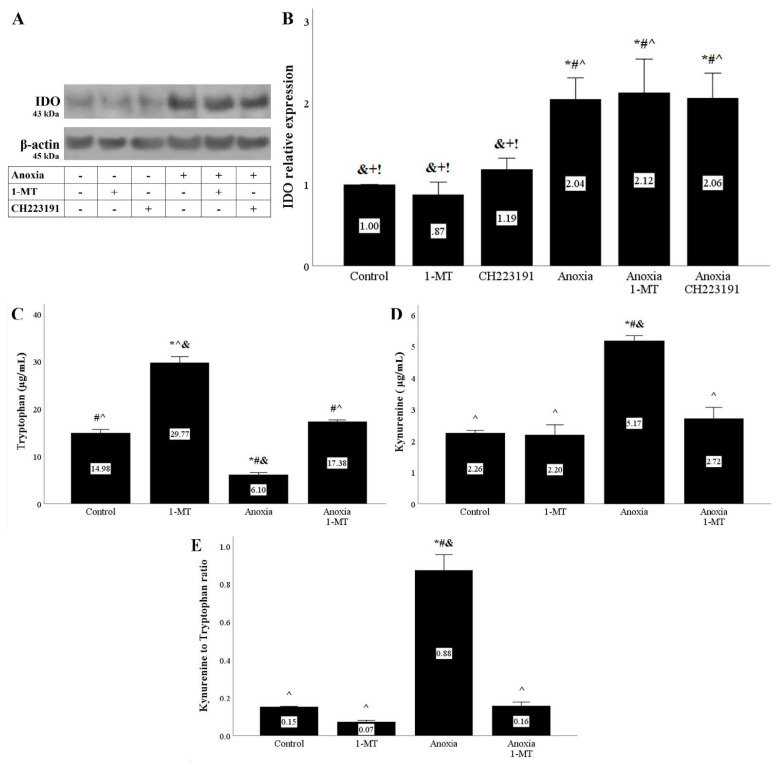
**Anoxia upregulates IDO expression and activity.** Cells were cultured under anoxic conditions for 24 h along with the indicating treatments. Anoxia in the presence or not of 1MT or CH223191 increased IDO protein level. A representative Western blot experiment is displayed in panel (**A**), whereas panel (**B**) depicts the cumulative results of four repeated experiments. * *p* < 0.05 vs. Control, ^#^ *p* < 0.05 vs. Control with 1-MT, ^ *p* < 0.05 vs. Control with CH223191, ^&^ *p* < 0.05 vs. Anoxia, ^+^ *p* < 0.05 vs. Anoxia with 1-MT, ^!^ *p* < 0.05 vs. Anoxia with CH223191. IDO activity assessed by the ratio of kynurenine to tryptophan concentration in cell culture supernatants also increased under anoxic conditions, an effect that is abolished by the IDO inhibitor 1-MT (**C**–**E**). Six such experiments were performed. * *p* < 0.05 vs. Control, ^#^ *p* < 0.05 vs. Control with 1-MT, ^ *p* < 0.05 vs. Anoxia, ^&^ *p* < 0.05 vs. Anoxia with 1-MT. Error bars correspond to SEM. 1-MT, 1-DL-methyltryptophan; IDO, indoleamine 2,3-dioxygenase 1.

**Figure 2:**
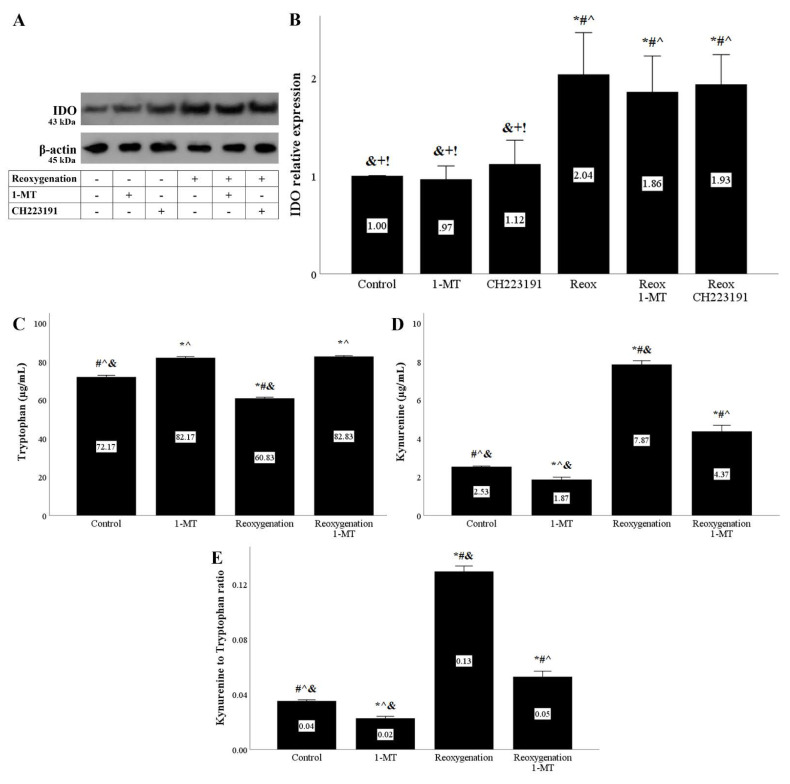
**Reoxygenation upregulates IDO expression and activity.** Cells were cultured under anoxic conditions for 24 h and then subjected to 2 h of reoxygenation along with the indicating treatments. Reoxygenation in the presence or not of 1-MT or CH223191 increased IDO protein level. A representative Western blot experiment is displayed in panel (**A**), whereas panel (**B**) depicts the cumulative results of four repeated experiments. * *p* < 0.05 vs. Control, ^#^ *p* < 0.05 vs. Control with 1-MT, ^ *p* < 0.05 vs. Control with CH223191, ^&^ *p* < 0.05 vs. Reoxygenation, ^+^ *p* < 0.05 vs. Reoxygenation with 1-MT, ^!^ *p* < 0.05 vs. Reoxygenation with CH223191. IDO activity assessed by the ratio of kynurenine to tryptophan concentration in cell culture supernatants also increased under reoxygenation, an effect that is abolished by the IDO inhibitor 1-MT (**C**–**E**). Six such experiments were performed. * *p* < 0.05 vs. Control, ^#^ *p* < 0.05 vs. Control with 1-MT, ^ *p* < 0.05 vs. Reoxygenation, ^&^ *p* < 0.05 vs. Reoxygenation with 1-MT. Error bars correspond to SEM. 1-MT, 1-DL-methyltryptophan; IDO, indoleamine 2,3-dioxygenase 1; Reox, reoxygenation.

Both anoxia and reoxygenation upregulated IDO levels in RPTECs. Neither the IDO inhibitor 1-MT nor the AhR inhibitor CH223191 affected IDO expression (Figure 1A and Figure 2A,B). 

The increase in IDO expression by anoxia or reoxygenation was also accompanied by elevated activity. Tryptophan concentration decreased, whereas kynurenine expression increased in the cell culture supernatants of RPTECs under anoxia or reoxygenation. The ratio of kynurenine to tryptophan concentration, which corresponds to IDO activity, increased under anoxia or reoxygenation as well. 1-MT successfully inhibited IDO activity (Figure 1C–E and Figure 2C–E) 

### 3.2. Anoxia and Reoxygenation Activate Both IDO Activity-Derived Pathways 

**Figure 3:**
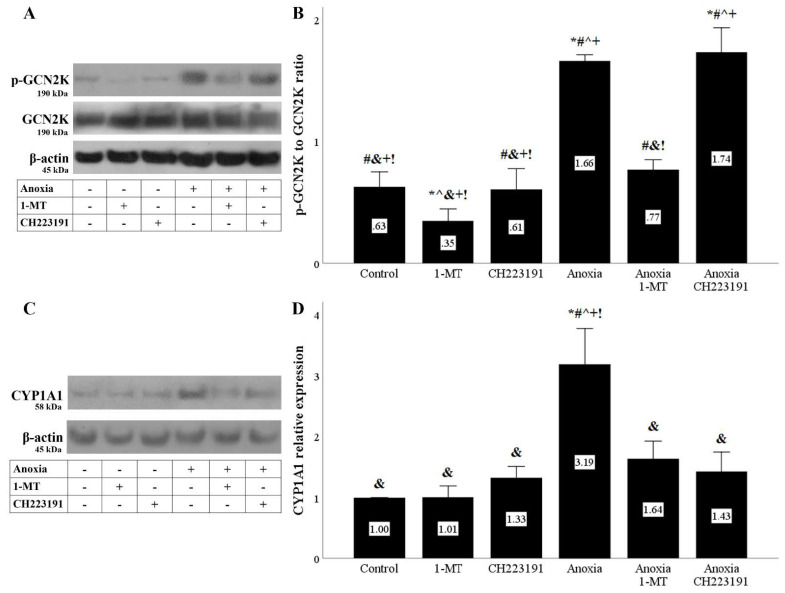
**Anoxia activates both IDO activity-derived pathways.** Cells were cultured under anoxic conditions for 24 h along with the indicating treatments. Anoxia activated the GCN2K pathway assessed by the level of phosphorylated activated GCN2K. 1-MT, but not CH223191, prevented GCN2K activation. A representative Western blot experiment is displayed in panel (**A**), whereas panel (**B**) depicts the cumulative results of four repeated experiments. Anoxia activated the AhR pathway assessed by the level of the AhR transcriptional target CYP1A1. Both the IDO inhibitor 1-MT and the AhR inhibitor CH223191 prevented AhR activation. A representative Western blot experiment is displayed in panel (**C**), whereas panel (**D**) depicts the cumulative results of four repeated experiments. * *p* < 0.05 vs. Control, ^#^ *p* < 0.05 vs. Control with 1-MT, ^ *p* < 0.05 vs. Control with CH223191, ^&^ *p* < 0.05 vs. Anoxia, ^+^ *p* < 0.05 vs. Anoxia with 1-MT, ^!^ *p* < 0.05 vs. Anoxia with CH223191. Error bars correspond to SEM. 1-MT, 1-DL-methyltryptophan; CYP1A1, cytochrome P450, family 1, subfamily A, polypeptide 1; GCN2K, general control nonderepressible 2 kinase; p-GCN2K, phosphorylated GCN2K.

**Figure 4:**
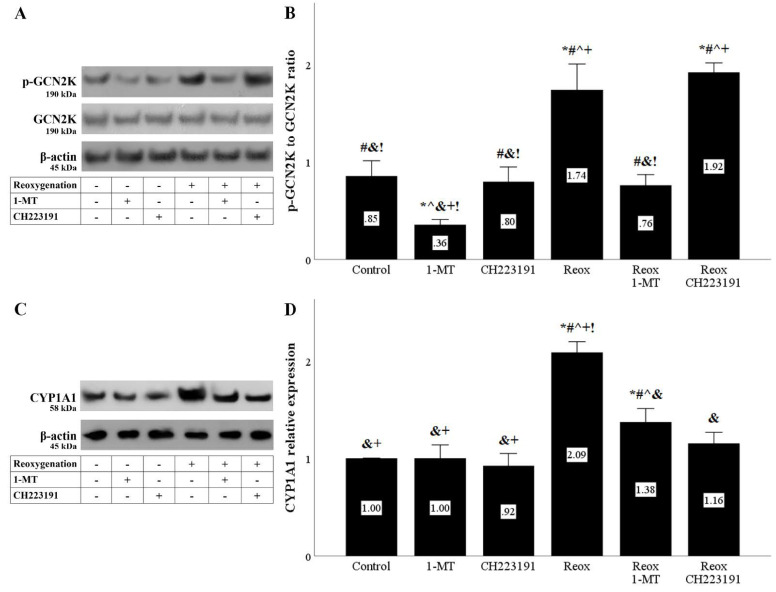
**Reoxygenation activates both IDO activity-derived pathways.** Cells were cultured under anoxic conditions for 24 h and then subjected to 2 h of reoxygenation along with the indicating treatments. Reoxygenation activated the GCN2K pathway assessed by the level of phosphorylated activated GCN2K. 1-MT, but not CH223191, prevented GCN2K activation. A representative Western blot experiment is displayed in panel (**A**), whereas panel (**B**) depicts the cumulative results of four repeated experiments. Reoxygenation activated the AhR pathway assessed by the level of the AhR transcriptional target CYP1A1. Both the IDO inhibitor 1-MT and the AhR inhibitor CH223191 prevented AhR activation. A representative Western blot experiment is displayed in panel (**C**), whereas panel (**D**) depicts the cumulative results of four repeated experiments. * *p* < 0.05 vs. Control, ^#^ *p* < 0.05 vs. Control with 1-MT, ^ *p* < 0.05 vs. Control with CH223191, ^&^ *p* < 0.05 vs. Reox, ^+^ *p* < 0.05 vs. Reox with 1-MT, ^!^ *p* < 0.05 vs. Reox with CH223191. Error bars correspond to SEM. 1-MT, 1-DL-methyltryptophan; CYP1A1, cytochrome P450, family 1, subfamily A, polypeptide 1; GCN2K, general control nonderepressible 2 kinase; p-GCN2K, phosphorylated GCN2K; Reox, reoxygenation.

The enhanced IDO activity in RPTECs cultured under anoxia or reoxygenation resulted in GCN2K activation assessed by the level of its activated phosphorylated form. As expected by inhibiting IDO, 1-MT inhibited GCN2K activation. On the contrary, the AhR inhibitor CH223191 had no effect on the GCN2K activation status (Figure 3A,B and Figure 4A,B).

Besides activation of the GCN2K pathway, the elevated IDO activity in RPTECs cultured under anoxia or reoxygenation also activated the AhR pathway assessed by the levels of the AhR transcriptional target CYP1A1. 1-MT inhibited AhR activation, and as expected, the same was observed in the case of RPTECs treated with the AhR inhibitor CH223191 (Figure 3C,D and Figure 4C,D).

### 3.3. Anoxia and Reoxygenation Induce DNA Damage Response That Is Ameliorated by Inhibition of IDO

**Figure 5:**
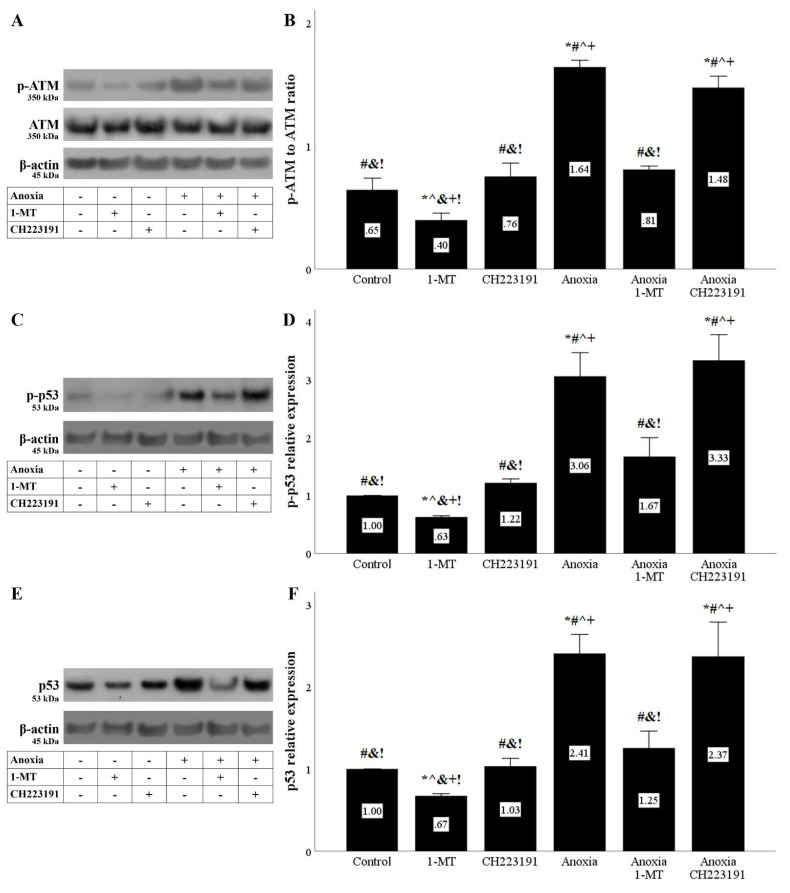
**Anoxia induces DNA damage response that is ameliorated by inhibition of IDO.** Cells were cultured under anoxic conditions for 24 h along with the indicating treatments. Anoxia induced a DNA damage response assessed by the level of phosphorylated activated ATM. 1-MT, but not CH223191, prevented p-ATM upregulation. A representative Western blot experiment is displayed in panel (**A**), whereas panel (**B**) depicts the cumulative results of four repeated experiments. Anoxia increased p-p53 level. 1-MT, but not CH223191, prevented p-p53 increase. A representative Western blot experiment is displayed in panel (**C**), whereas panel (**D**) depicts the cumulative results of four repeated experiments. Anoxia increased p53 level. 1-MT, but not CH223191, prevented p53 rise. A representative Western blot experiment is displayed in panel (**E**), whereas panel (**F**) depicts the cumulative results of four repeated experiments. * *p* < 0.05 vs. Control, ^#^ *p* < 0.05 vs. Control with 1-MT, ^ *p* < 0.05 vs. Control with CH223191, ^&^ *p* < 0.05 vs. Anoxia, ^+^ *p* < 0.05 vs. Anoxia with 1-MT, ^!^ *p* < 0.05 vs. Anoxia with CH223191. Error bars correspond to SEM. 1-MT, 1-DL-methyltryptophan; p-p53, phosphorylated p53.

**Figure 6:**
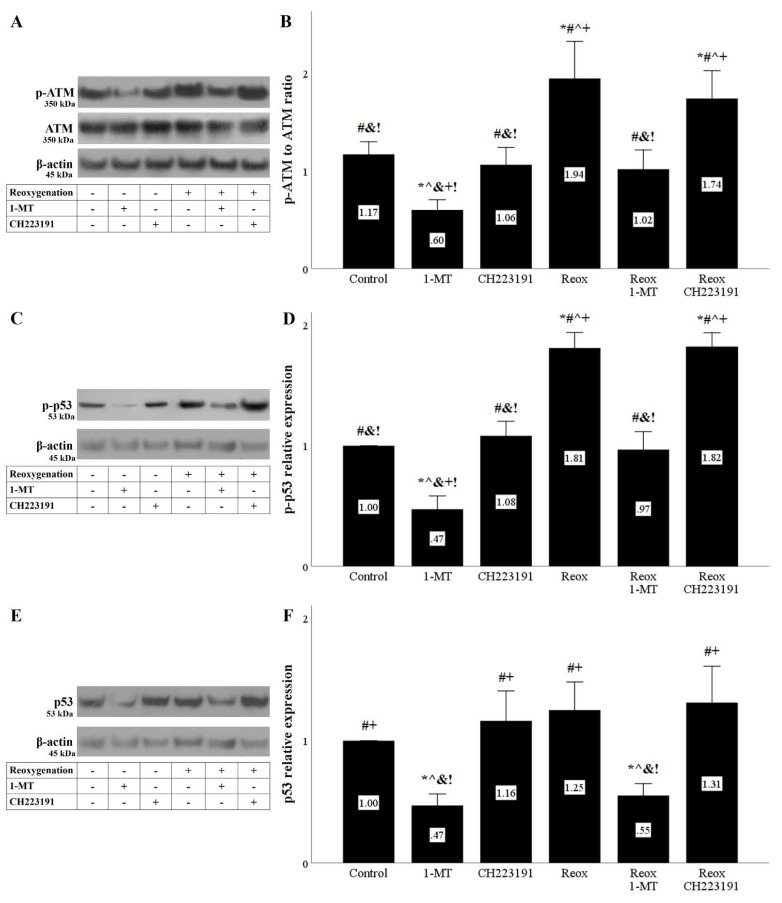
**Reoxygenation induces DNA damage response that is ameliorated by inhibition of IDO.** Cells were cultured under anoxic conditions for 24 h and then subjected to 2 h of reoxygenation along with the indicating treatments. Reoxygenation induced a DNA damage response assessed by the level of phosphorylated activated ATM. 1-MT, but not CH223191, prevented p-ATM upregulation. A representative Western blot experiment is displayed in panel (**A**), whereas panel (**B**) depicts the cumulative results of four repeated experiments. Reoxygenation increased p-p53 level. 1-MT, but not CH223191, prevented p-p53 increase. A representative Western blot experiment is displayed in panel (**C**), whereas panel (**D**) depicts the cumulative results of four repeated experiments. Although 1-MT decreased p53 levels, reoxygenation per se did not affect p53. CH223191 left p53 levels unaffected. A representative Western blot experiment is displayed in panel (**E**), whereas panel (**F**) depicts the cumulative results of four repeated experiments. * *p* < 0.05 vs. Control, ^#^ *p* < 0.05 vs. Control with 1-MT, ^ *p* < 0.05 vs. Control with CH223191, ^&^ *p* < 0.05 vs. Reox, ^+^ *p* < 0.05 vs. Reox with 1-MT, ^!^ *p* < 0.05 vs. Reox with CH223191. Error bars correspond to SEM. 1-MT, 1-DL-methyltryptophan; p-p53, phosphorylated p53, Reox, reoxygenation.

Anoxia or reoxygenation induced the initiation of DDR assessed by the level of activated phosphorylated ATM. Inhibition of IDO with 1-MT ameliorated the DDR, whereas the AhR inhibitor CH223191 had no effect (Figure 5A,B and Figure 6A,B).

As expected, the activated ATM phosphorylated its substrate p53. The levels of p-p53 were elevated during anoxia or reoxygenation. 1-MT reduced p-p53, whereas CH223191 did not affect the p-p53 level (Figure 5C,D and Figure 6C,D).

Since p53 phosphorylation protects it from proteasomal degradation, the fact that under anoxia total p53 levels were increased was expected. 1-MT reduced the total p53 level, whereas CH223191 did not have any effect on total p53 levels (Figure 5E,F). However, and despite its impact on p-p53, reoxygenation did not enhance the total p53 level. Yet, the IDO inhibitor 1-MT decreased total p53, whereas the AhR inhibitor CH223191 did not affect total p53 levels (Figure 6E,F).

### 3.4. Anoxia and Reoxygenation Upregulate the Cell-Cycle Inhibitors p21 and p16, Whereas Inhibition of IDO Prevents Their Upregulation

**Figure 7:**
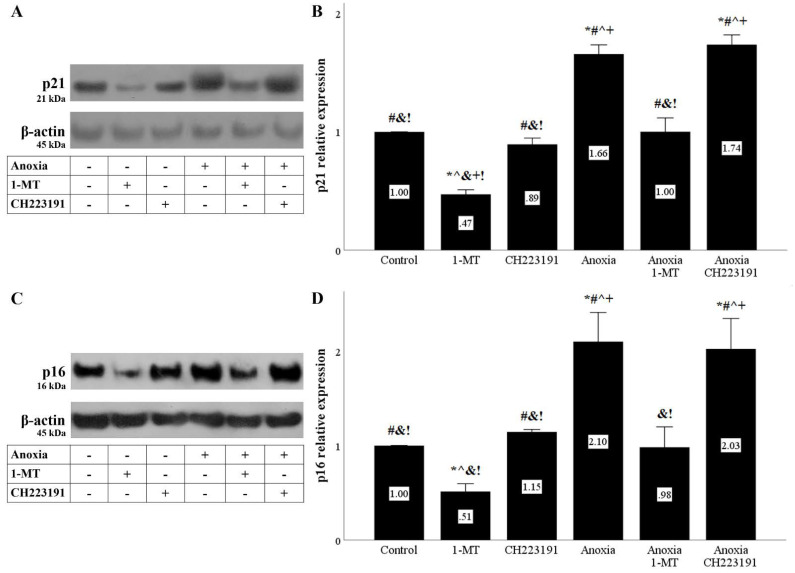
**Anoxia upregulates the cell-cycle inhibitors p21 and p16, whereas inhibition of IDO prevents their upregulation.** Cells were cultured under anoxic conditions for 24 h along with the indicating treatments. Anoxia upregulated p21. 1-MT, but not CH223191, prevented p21 upregulation. A representative Western blot experiment is displayed in panel (**A**), whereas panel (**B**) depicts the cumulative results of four repeated experiments. Anoxia increased p16 levels. 1-MT, but not CH223191, prevented p16 increase. A representative Western blot experiment is displayed in panel (**C**), whereas panel (**D**) depicts the cumulative results of four repeated experiments. * *p* < 0.05 vs. Control, ^#^ *p* < 0.05 vs. Control with 1-MT, ^ *p* < 0.05 vs. Control with CH223191, ^&^ *p* < 0.05 vs. Anoxia, ^+^ *p* < 0.05 vs. Anoxia with 1-MT, ^!^ *p* < 0.05 vs. Anoxia with CH223191. Error bars correspond to SEM. 1-MT, 1-DL-methyltryptophan.

**Figure 8:**
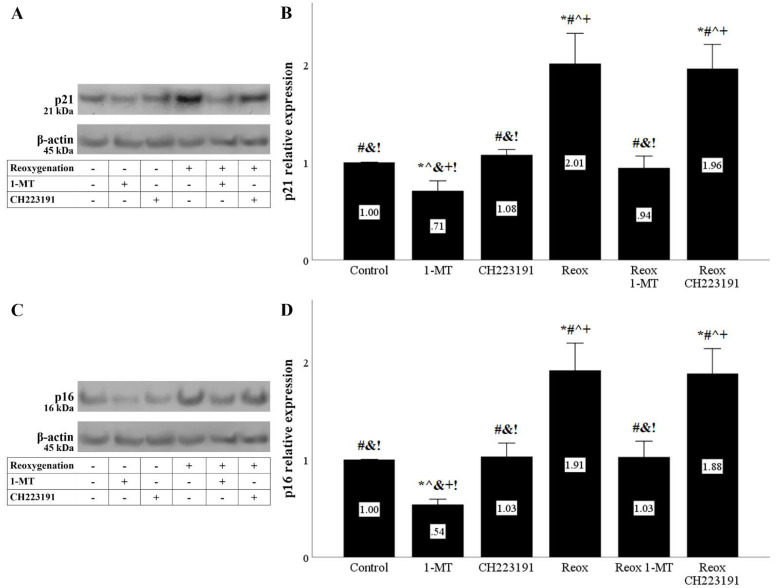
Reoxygenation upregulates the cell-cycle inhibitors p21 and p16, whereas inhibition of IDO prevents their upregulation. Cells were cultured under anoxic conditions for 24 h and then subjected to 2 h of reoxygenation along with the indicating treatments. Reoxygenation increased p21 levels. 1-MT, but not CH223191, prevented p21 increase. A representative Western blot experiment is displayed in panel (**A**), whereas panel (**B**) depicts the cumulative results of four repeated experiments. Reoxygenation upregulated p16 levels. 1-MT, but not CH223191, prevented p16 upregulation. A representative Western blot experiment is displayed in panel (**C**), whereas panel (**D**) depicts the cumulative results of four repeated experiments. * *p* < 0.05 vs. Control, ^#^ *p* < 0.05 vs. Control with 1-MT, ^ *p* < 0.05 vs. Control with CH223191, ^&^ *p* < 0.05 vs. Reox, ^+^ *p* < 0.05 vs. Reox with 1-MT, ^!^ *p* < 0.05 vs. Reox with CH223191. Error bars correspond to SEM. 1-MT, 1-DL-methyltryptophan; Reox, reoxygenation.

Both anoxia and reoxygenation increased the cell-cycle inhibitor p21. Inhibition of IDO reduced p21, whereas inhibition of AhR did not induce any change in p21 levels (Figure 7A,B and Figure 8A,B).

The cell-cycle inhibitor p16 was also upregulated under anoxia or reoxygenation. 1-MT decreased p16, whereas CH223191 did not affect p16 levels (Figure 7C,D and Figure 8C,D). 

### 3.5. Anoxia and Reoxygenation Upregulate SA-β-Gal Activity, Whereas Inhibition of IDO Prevents Its Upregulation

**Figure 9:**
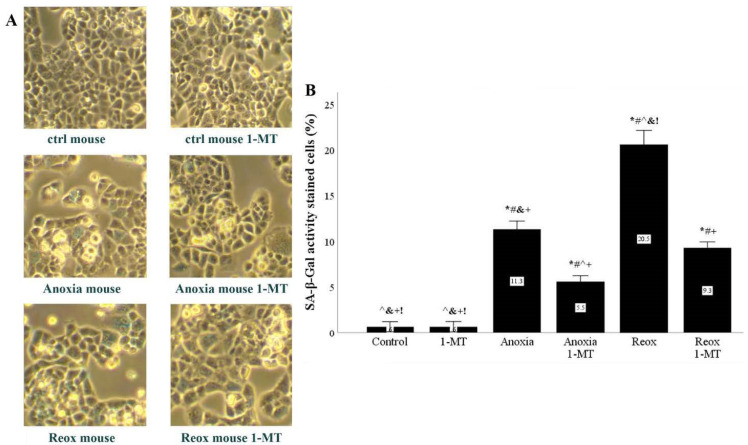
**In mouse RPTECS, IDO inhibition decreased anoxia- or reoxygenation-induced upregulation of SA-β-Gal activity.** Mouse RPTECs were cultured under anoxic conditions for 24 h or subsequently subjected to reoxygenation for another 2 h. A representative of three independent experiments is depicted in panel (**A**). Anoxia upregulated SA-β-Gal activity, which was further upregulated by reoxygenation. In both cases, the IDO inhibitor 1-MT decreased SA-β-Gal activity (**B**). * *p* < 0.05 vs. Control, ^#^ *p* < 0.05 vs. Control with 1-MT, ^ *p* < 0.05 vs. Anoxia, ^&^ *p* < 0.05 vs. Anoxia with 1-MT, ^+^ *p* < 0.05 vs. Reox, ^!^ *p* < 0.05 vs. Reox with 1-MT. Error bars correspond to SEM. 1-MT, 1-DL-methyltryptophan; Reox, reoxygenation; SA-β-Gal, senescence-associated β-galactosidase.

In mouse RPTECs, histochemistry detected that anoxia upregulates SA-β-Gal activity, which was upregulated further by the subsequent reoxygenation. In both cases, inhibition of IDO decreased SA-β-Gal activity (Figure 9A,B).

### 3.6. Anoxia and Reoxygenation Inhibit Cell Proliferation and Induce IL-6 Production in An IDO Dependent Manner

**Figure 10:**
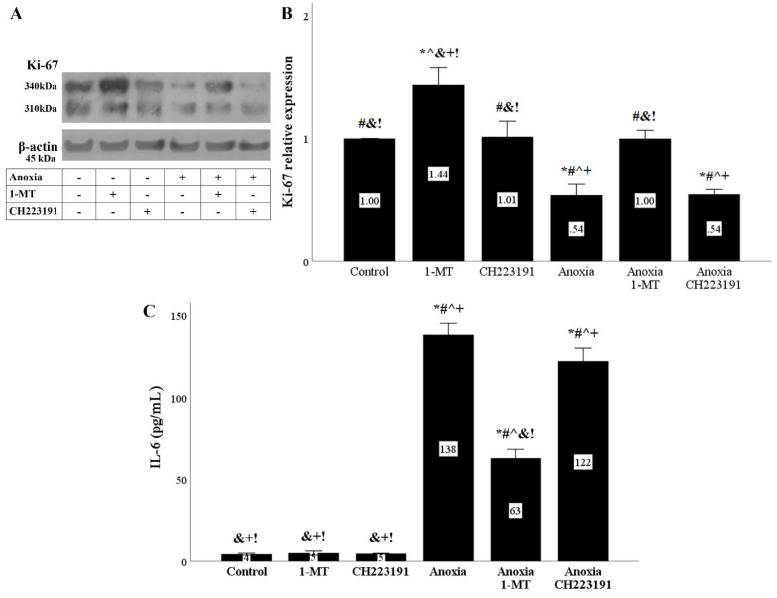
**Anoxia inhibits cell proliferation and induces IL-6 production in an IDO dependent manner.** Cells were cultured under anoxic conditions for 24 h along with the indicating treatments. Anoxia decreased the levels of the cell proliferation marker Ki-67. The IDO inhibitor 1-MT restored Ki-67 levels, whereas the AhR inhibitor CH223191 left Ki-67 levels unaffected. A representative Western blot experiment is displayed in panel (**A**), whereas panel (**B**) depicts the cumulative results of four repeated experiments. Anoxia increased IL-6 concentration in the RPTECs culture supernatants. 1-MT decreased the anoxia-induced IL-6 production, whereas CH223191 did not affect IL-6 levels (**C**). Six such experiments were performed. * *p* < 0.05 vs. Control, ^#^ *p* < 0.05 vs. Control with 1-MT, ^ *p* < 0.05 vs. Control with CH223191, ^&^ *p* < 0.05 vs. Anoxia, ^+^ *p* < 0.05 vs. Anoxia with 1-MT, ^!^ *p* < 0.05 vs. Anoxia with CH223191. Error bars correspond to SEM. 1-MT, 1-DL-methyltryptophan; IL-6, interleukin-6.

**Figure 11:**
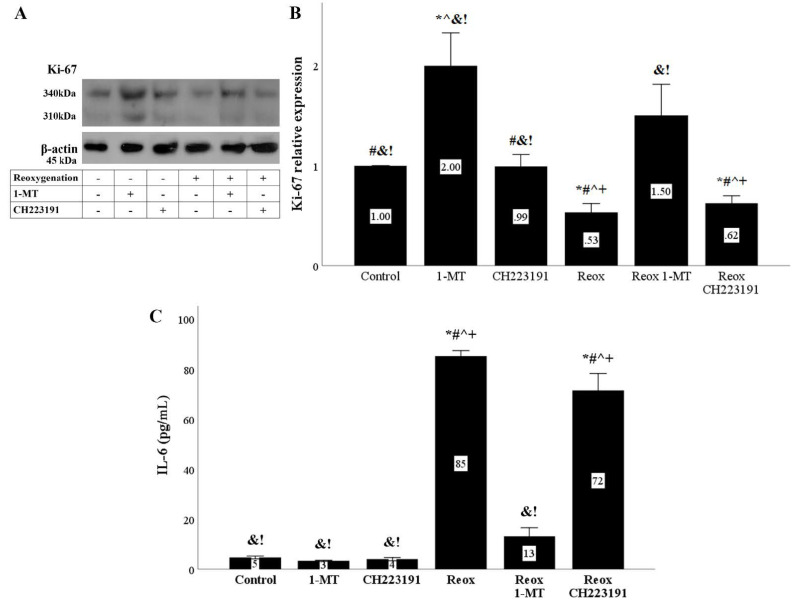
**Reoxygenation inhibits cell proliferation and induces IL-6 production in an IDO dependent manner.** Cells were cultured under anoxic conditions for 24 h and then subjected to 2 h of reoxygenation along with the indicating treatments. Reoxygenation reduced the levels of the cell proliferation marker Ki-67. The IDO inhibitor 1-MT restored Ki-67 levels, whereas the AhR inhibitor CH223191 did not affect Ki-67. A representative Western blot experiment is displayed in panel (**A**), whereas panel (**B**) depicts the cumulative results of four repeated experiments. Reoxygenation increased IL-6 concentration in the RPTECs culture supernatants. 1-MT reduced the reoxygenation-induced IL-6 production, whereas CH223191 did not affect IL-6 levels (**C**). Six such experiments were performed. * *p* < 0.05 vs. Control, ^#^ *p* < 0.05 vs. Control with 1-MT, ^ *p* < 0.05 vs. Control with CH223191, ^&^ *p* < 0.05 vs. Reox, ^+^ *p* < 0.05 vs. Reox with 1-MT, ^!^ *p* < 0.05 vs. Reox with CH223191. Error bars correspond to SEM. 1-MT, 1-DL-methyltryptophan; IL-6, interleukin-6; Reox, reoxygenation.

RPTECs proliferation assessed by the proliferation marker Ki-67 was inhibited under anoxia or reoxygenation. IDO inhibition by 1-MT restored Ki-67 levels. On the contrary, the AhR inhibitor CH223191 did not alter Ki-67 levels (Figure 10A,B and Figure 11A,B). 

Both anoxia and reoxygenation induced IL-6 production by RPTECs. 1-MT inhibited IL-6 production, whereas CH223191 did not have any effect on IL-6 production (Figure 9C and Figure 10C).

### 3.7. In Human RPTECs, Anoxia and Reoxygenation Also Upregulate SA-β-Gal Activity, Whereas Inhibition of IDO Prevents Its Upregulation

**Figure 12:**
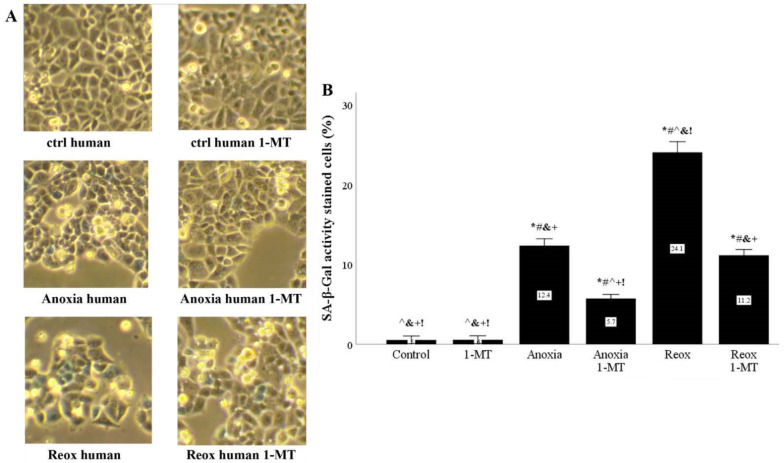
**In human RPTECS, inhibition of IDO reduces anoxia- or reoxygenation-induced upregulation of SA-β-Gal activity.** Primary human RPTECs were cultured under anoxia for 2 h or subjected to reoxygenation for another 24 h. A representative of three independent experiments is depicted in panel (**A**). Anoxia increased SA-β-Gal activity, which was further increased by reoxygenation. The IDO inhibitor 1-MT reduced both anoxia and reoxygenation-induced upregulation of SA-β-Gal activity (**B**). * *p* < 0.05 vs. Control, ^#^ *p* < 0.05 vs. Control with 1-MT, ^ *p* < 0.05 vs. Anoxia, ^&^ *p* < 0.05 vs. Anoxia with 1-MT, ^+^ *p* < 0.05 vs. Reox, ^!^ *p* < 0.05 vs. Reox with 1-MT. Error bars correspond to SEM. 1-MT, 1-DL-methyltryptophan; Reox, reoxygenation; SA-β-Gal, senescence-associated β-galactosidase.

Histochemistry revealed that in human RPTECs, anoxia increased SA-β-Gal activity, which was increased further by the following reoxygenation. In both cases, the IDO inhibitor 1-MT reduced SA-β-Gal activity (Figure 12A,B).

## 4. Discussion

In I-R-induced AKI, cellular senescence contributes to incomplete recovery of kidney function by preventing renal tubular cell dedifferentiation and proliferation [8,9,11]. In parallel, cellular senescence has been incriminated in the subsequent development of CKD [8,9,11]. Patients who survive AKI are nine times more likely to develop CKD and have three times increased probability of end-stage renal disease [25,26]. The so-called senescence-associated secretory phenotype of the senescent renal tubular epithelial cells by promoting inflammation and fibrosis is thought to be responsible for progression to end-stage renal disease [8,9,11]. Thus, studying the biology of I-R-induced renal tubular cell senescence is important and may lead to novel preventive or ameliorative therapeutic maneuvers.

In renal tubular epithelial cells, IDO is upregulated during I-R injury and detrimentally affects the outcome regarding cell survival [20,21]. However, the role of IDO in the senescence of renal tubular epithelial cells has not yet been evaluated. This was the aim of our study. Since tryptophan is an essential amino acid not synthesized by human cells, with the lowest concentration among all the amino acids, IDO could be considered a potential and sensitive sensor of cellular stress. Remarkably, in humans, only a two-day low tryptophan intake suffices for tryptophan depletion [27]. 

In accordance with previous studies [20,21], we detected that both anoxia and reoxygenation upregulate IDO expression in RPTECs. In parallel to the increased IDO expression, IDO activity assessed by the ratio of kynurenine to tryptophan concentration was also enhanced. The IDO inhibitor 1-MT successfully suppressed IDO activity, indicating that under the concentration used in our experiments, 1-MT was effective. 

Anoxia and reoxygenation by depleting tryptophan activated GCN2K. Depletion of tryptophan results in uncharged tryptophanyl-tRNA, which alters GCN2K conformation, allowing for its autophosphorylation and activation. In turn, activated GCN2K phosphorylates and activates eukaryotic translation initiation factor-2α (eIF2α), changing the translational program of the cell [13,14]. As expected, the IDO inhibitor 1-MT decreased the anoxia or reoxygenation-induced GCN2K activation.

Anoxia and reoxygenation, by promoting kynurenine production, activate the transcription factor AhR, as indicated by the upregulation of its transcriptional target CYP1A1 [18,19]. AhR has many transcriptional targets, and CYP1A1 contributes to ROS overproduction during reoxygenation and cell death through ferroptosis [21,24]. Both the IDO inhibitor 1-MT, by decreasing kynurenine production, and the AhR inhibitor CH223191 prevented CYP1A1 upregulation.

Anoxia and reoxygenation induced DDR, as detected by the level of phosphorylated ATM. ATM is a serine/threonine protein kinase that senses DNA double-strand breaks, and then it is autophosphorylated and activated [28]. Inhibition of AhR did not affect the DDR, whereas 1-MT ameliorated DDR significantly. The latter indicates that from the two IDO activity-derived pathways, only the pathway of GCN2K activation affects the DDR. In contrast, the AhR activation pathway does not affect the DDR. A possible mechanism for the enhancement of the DDR by IDO-induced GCN2K activation relies on the ability of GCN2K to phosphorylate methionyl-tRNA synthetase (MRS). MRS forms a complex with aminoacyl-tRNA synthetase-interacting multifunctional protein-3/p18 (AIMP3/p18), preventing its entry into the nucleus. When MRS gets phosphorylated by GCN2K, AIMP3/p18 is released, translocates into the nucleus, and forms a complex with ATM/Rad3-related protein (ATR), a prerequisite for ATM activation and initiation of the DDR [16,17]. The above-described pathway may also explain our observations concerning the effect of 1-MT in many of the evaluated parameters, even in RPTECs cultured under normoxic conditions.

One ATM/ATR target is the tumor suppressor p53 [28,29]. Once phosphorylated by the ATM/ATR, p53 dissociates the E3 ubiquitin-protein ligase Mdm2 and escapes proteasomal degradation, resulting in its accumulation [29]. As expected, by activating ATM/ATR, anoxia and reoxygenation increased the level of p-p53.1-MT, which reduced the DDR, inhibited the phosphorylation of p53. On the contrary, CH223101 left p53 phosphorylation status unaffected. Unsurprisingly, in anoxia, total p53 levels followed the increased trend of its phosphorylated form. It decreased by 1-MT and remained unaffected by CH223191. Although 1-MT decreased total p53 under reoxygenation, reoxygenation per se did not enhance total p53 level. This indicates the complex nature of p53 regulation [29,30] and may also explain the observation that renal tubular epithelial cells die by apoptosis during anoxia and by ferroptosis during reoxygenation [4,5,6]. The tumor suppressor p53 transcribes many pro-apoptotic genes [29], whereas under certain circumstances, its transcriptional target p21 may downregulate p53 stability [30] or inhibit apoptosis [31]. 

Anoxia, by increasing p53, upregulated its transcriptional target p21 [29]. This well-known cell-cycle inhibitor can induce both G1/S and G2/M cell-cycle arrest by inhibiting cyclin-dependent kinase (CDK)4,6/cyclin-D and CDK2/cyclin-E complexes, respectively [32]. 1-MT downregulated p21, whereas CH223191 did not affect p21 level since it did not affect p53 as well. Reoxygenation also upregulated p21, an effect that was abolished by 1-MT but not by CH223191. The fact that under reoxygenation, p21 was upregulated without a concurrent p53 increase indicates that a p53-independent DDR-induced mechanism of p21 upregulation may be implicated [32], or simply that p21 remains increased from the previous anoxic phase.

The cell-cycle inhibitor p21 is considered a marker of senescence [33], and studies have shown that it contributes to senescence initiation [34,35]. However, p16 is generally considered a better marker of senescence [33], which can induce G1/S cell-cycle arrest by inhibiting CDK4,6/cyclin D complex, resulting in a hypo-phosphorylated retinoblastoma protein (pRB) state that keeps the S-phase initiating transcription factor E2F inactive [34,35]. This cell-cycle inhibitor is considered responsible for maintaining the cell in the senescent state [34,35]. During the DDR, activated ATM phosphorylates the histone-lysine N-methyltransferase enzyme enhancer of zeste homolog 2 (EZH2), leading to its rapid degradation. As a consequence of less trimethylation at lysine 27 histone H3 (H3K27me3), the transcription of p16 and various cytokines is upregulated [36]. In our experiments, both anoxia and reoxygenation, by inducing the DDR and activating ATM, increased p16. 1-MT, by ameliorating the DDR, decreased p16 level, whereas CH223191 left p16 unaffected.

The beneficial role of IDO inhibition in ameliorating anoxia- or reoxygenation-induced cellular senescence in mouse RPTECs was also confirmed histochemically by assessing the senescence marker SA-β-Gal [33]. Anoxia or reoxygenation increased the activity of SA-β-Gal, whereas the IDO inhibitor 1-MT decreased it.

To define whether the anoxia- or reoxygenation-induced upregulation of p21 and p16 affects cell proliferation, we evaluated the level of the cell-proliferation marker Ki-67 [37]. Indeed, both anoxia and reoxygenation decreased Ki-67. As expected by the effect of 1-MT on the cell-cycle inhibitors, the IDO inhibitor ameliorated inhibition of cell proliferation, whereas CH223191 had no effect. 

Another phenotypical characteristic of senescent cells is the production of proinflammatory and profibrotic cytokines [8,9,11]. The activated by DNA-damage ATM blocks the p62-dependent autophagic degradation of the transcription factor GATA4. Accumulated GATA4 induces tumor necrosis factor receptor-associated factor interacting protein 2 (TRAF3IP2) and interleukin 1A (IL-1A), which activates NF-kB to initiate and maintain the senescence-associated secretory phenotype [38]. We evaluated the production of the proinflammatory IL-6 under our experimental conditions. Both anoxia and reoxygenation increased IL-6 production. As expected, in both cases, 1-MT decreased IL-6 upregulation, whereas CH223191 had no effect on IL-6.

Most of the study experiments were performed in mouse RPTECs since we aimed to extend our research toward an in vivo experimental model of murine kidney I-R-induced senescence. However, since clinical application is the final target of the research, we also evaluated whether inhibition of IDO ameliorates anoxia- or reoxygenation-induced senescence in primary human RPTECs. Indeed, we detected that anoxia or reoxygenation increased the activity of SA-β-Gal in human RPTECs, whereas the IDO inhibitor 1-MT decreased it. 

The in vitro nature of our study is a limitation. However, since the role of IDO on I-R injury-induced renal senescence has never been evaluated before, our study could be considered a starting point. Interestingly, IDO inhibitors have already been tested in clinical trials on cancer immunotherapy, and much is known about their pharmacokinetics and safety [39]. In addition, as already noted, I-R injury consists of two consecutive but pathophysiologically distinct phases. Cell culture is ideal for evaluating the effect of IDO on RPTEC senescence during these two different phases of I-R injury. 

In conclusion, anoxia and the subsequent reoxygenation upregulate IDO. IDO depletes tryptophan and activates GCN2K. The latter enhances the anoxia- or reoxygenation-induced DDR, resulting in increased p21 and p16 expression and eventually in RPTEC senescence. Since cellular senescence contributes to incomplete recovery after I-R-induced AKI and predisposes to subsequent CKD, the role of IDO in cellular senescence and the possible therapeutic role of IDO inhibitors deserve further investigation.

## Data Availability

The analyzed datasets generated during the study are available from the corresponding author on reasonable request.

## References

[1] Uchino S., Kellum J.A., Bellomo R., Doig G.S., Morimatsu H., Morgera S., Schetz M., Tan I., Bouman C., Macedo E. (2005). Acute renal failure in critically ill patients: A multinational, multicenter study. JAMA.

[2] Bonventre J.V., Yang L. (2011). Cellular pathophysiology of ischemic acute kidney injury. J. Clin. Investig..

[3] Wu M.Y., Yiang G.T., Liao W.T., Tsai A.P., Cheng Y.L., Cheng P.W., Li C.Y., Li C.J. (2018). Current Mechanistic Concepts in Ischemia and Reperfusion Injury. Cell. Physiol. Biochem..

[4] Eleftheriadis T., Pissas G., Antoniadi G., Liakopoulos V., Stefanidis I. (2018). Cell Death Patterns Due to Warm Ischemia or Reperfusion in Renal Tubular Epithelial Cells Originating from Human, Mouse, or the Native Hibernator Hamster. Biology.

[5] Eleftheriadis T., Pissas G., Liakopoulos V., Stefanidis I. (2019). Factors that May Protect the Native Hibernator Syrian Hamster Renal Tubular Epithelial Cells from Ferroptosis Due to Warm Anoxia-Reoxygenation. Biology.

[6] Linkermann A., Skouta R., Himmerkus N., Mulay S.R., Dewitz C., De Zen F., Prokai A., Zuchtriegel G., Krombach F., Welz P.S. (2014). Synchronized renal tubular cell death involves ferroptosis. Proc. Natl. Acad. Sci. USA.

[7] Hsu C.Y. (2012). Yes, AKI truly leads to CKD. J. Am. Soc. Nephrol..

[8] Canaud G., Bonventre J.V. (2014). Cell cycle arrest and the evolution of chronic kidney disease from acute kidney injury. Nephrol. Dial. Transplant..

[9] Docherty M.-H., O’Sullivan E.D., Bonventre J.V., Ferenbach D.A. (2019). Cellular Senescence in the Kidney. J. Am. Soc. Nephrol..

[10] Yan M., Tang C., Ma Z., Huang S., Dong Z. (2016). DNA damage response in nephrotoxic and ischemic kidney injury. Toxicol. Appl. Pharmacol..

[11] Yu S.M.W., Bonventre J.V. (2020). Acute kidney injury and maladaptive tubular repair leading to renal fibrosis. Curr. Opin. Nephrol. Hypertens..

[12] Li C., Shen Y., Huang L., Liu C., Wang J. (2020). Senolytic therapy ameliorates renal fibrosis postacute kidney injury by alleviating renal senescence. FASEB J..

[13] Castilho B.A., Shanmugam R., Silva R.C., Ramesh R., Himme B.M., Sattlegger E. (2014). Keeping the eIF2 alpha kinase Gcn2 in check. Biochim. Biophys. Acta.

[14] Eleftheriadis T., Pissas G., Antoniadi G., Spanoulis A., Liakopoulos V., Stefanidis I. (2014). Indoleamine 2,3-dioxygenase increases p53 levels in alloreactive human T cells, and both indoleamine 2,3-dioxygenase and p53 suppress glucose uptake, glycolysis and proliferation. Int. Immunol..

[15] Mata J., Anda S., Zach R., Grallert B. (2017). Activation of Gcn2 in response to different stresses. PLoS ONE.

[16] Park B.J., Kang J.W., Lee S.W., Choi S.J., Shin Y.K., Ahn Y.H., Choi Y.H., Choi D., Lee K.S., Kim S. (2005). The haploinsufficient tumor suppressor p18 upregulates p53 via interactions with ATM/ATR. Cell.

[17] Kwon N.H., Kang T., Lee J.Y., Kim H.H., Kim H.R., Hong J., Oh Y.S., Han J.M., Ku M.J., Lee S.Y. (2011). Dual role of methionyl-tRNA synthetase in the regulation of translation and tumor suppressor activity of aminoacyl-tRNA synthetase-interacting multifunctional protein-3. Proc. Natl. Acad. Sci. USA.

[18] Mezrich J.D., Fechner J.H., Zhang X., Johnson B.P., Burlingham W.J., Bradfield C.A. (2010). An interaction between kynurenine and the aryl hydrocarbon receptor can generate regulatory T cells. J. Immunol..

[19] Stejskalova L., Dvorak Z., Pavek P. (2011). Endogenous and exogenous ligands of aryl hydrocarbon receptor: Current state of art. Curr. Drug. Metab..

[20] Mohib K., Wang S., Guan Q., Mellor A.L., Sun H., Du C., Jevnikar A.M. (2008). Indoleamine 2,3-dioxygenase expression promotes renal ischemia-reperfusion injury. Am. J. Physiol. Renal Physiol..

[21] Eleftheriadis T., Pissas G., Golfinopoulos S., Liakopoulos V., Stefanidis I. (2021). Role of indoleamine 2,3-dioxygenase in ischemia-reperfusion injury of renal tubular epithelial cells. Mol. Med. Rep..

[22] Jia L., Schweikart K., Tomaszewski J., Page J.G., Noker P.E., Buhrow S.A., Reid J.M., Ames M.M., Munn D.H. (2008). Toxicology and pharmacokinetics of 1-methyl-d-tryptophan: Absence of toxicity due to saturating absorption. Food Chem. Toxicol..

[23] Kim S.H., Henry E.C., Kim D.K., Kim Y.H., Shin K.J., Han M.S., Lee T.G., Kang J.K., Gasiewicz T.A., Ryu S.H. (2006). Novel compound 2-methyl-2H-pyrazole-3-carboxylic acid (2-methyl-4-o-tolylazo-phenyl)-amide (CH-223191) prevents 2,3,7,8-TCDD-induced toxicity by antagonizing the aryl hydrocarbon receptor. Mol. Pharmacol..

[24] Eleftheriadis T., Pissas G., Filippidis G., Liakopoulos V., Stefanidis I. (2020). Reoxygenation induces reactive oxygen species production and ferroptosis in renal tubular epithelial cells by activating aryl hydrocarbon receptor. Mol. Med. Rep..

[25] Wald R., Quinn R.R., Luo J., Li P., Scales D.C., Mamdani M.M., Ray J.G., University of Toronto Acute Kidney Injury Research Group (2009). Chronic dialysis and death among survivors of acute kidney injury requiring dialysis. JAMA.

[26] Coca S.G., Singanamala S., Parikh C.R. (2012). Chronic kidney disease after acute kidney injury: A systematic review and meta-analysis. Kidney Int..

[27] Ellenbogen M.A., Young S.N., Dean P., Palmour R.M., Benkelfat C. (1996). Mood response to acute tryptophan depletion in healthy volunteers: Sex differences and temporal stability. Neuropsychopharmacology.

[28] Rotman G., Shiloh Y. (1999). ATM: A mediator of multiple responses to genotoxic stress. Oncogene.

[29] Brady C.A., Attardi L.D. (2010). p53 at a glance. J. Cell Sci..

[30] Broude E.V., Demidenko Z.N., Vivo C., Swift M.E., Davis B.M., Blagosklonny M.V., Roninson I.B. (2014). p21 (CDKN1A) is a Negative Regulator of p53 Stability. Cell Cycle.

[31] Mirzayans R., Andrais B., Kumar P., Murray D. (2017). Significance of Wild-Type p53 Signaling in Suppressing Apoptosis in Response to Chemical Genotoxic Agents: Impact on Chemotherapy Outcome. Int. J. Mol. Sci..

[32] Karimian A., Ahmadi Y., Yousefi B. (2016). Multiple functions of p21 in cell cycle, apoptosis and transcriptional regulation after DNA damage. DNA Repair.

[33] Hernandez-Segura A., Nehme J., Demaria M. (2018). Hallmarks of Cellular Senescence. Trends Cell Biol..

[34] Rayess H., Wang M.B., Srivatsan E.S. (2012). Cellular senescence and tumor suppressor gene p16. Int. J. Cancer.

[35] Mijit M., Caracciolo V., Melillo A., Amicarelli F., Giordano A. (2020). Role of p53 in the Regulation of Cellular Senescence. Biomolecules.

[36] Ito T., Teo Y.V., Evans S.A., Neretti N., Sedivy J.M. (2018). Regulation of Cellular Senescence by Polycomb Chromatin Modifiers through Distinct DNA Damage- and Histone Methylation-Dependent Pathways. Cell Rep..

[37] Schlüter C., Duchrow M., Wohlenberg C., Becker M.H., Key G., Flad H.D., Gerdes J. (1993). The cell proliferation-associated antigen of antibody Ki-67: A very large, ubiquitous nuclear protein with numerous repeated elements, representing a new kind of cell cycle-maintaining proteins. J. Cell Biol..

[38] Kang C., Xu Q., Martin T.D., Li M.Z., Demaria M., Aron L., Lu T., Yankner B.A., Campisi J., Elledge S.J. (2015). The DNA damage response induces inflammation and senescence by inhibiting autophagy of GATA4. Science.

[39] Tang K., Wu Y.-H., Song Y., Yu B. (2021). Indoleamine 2,3-dioxygenase 1 (IDO1) inhibitors in clinical trials for cancer immunotherapy. J. Hematol. Oncol..

